# 3D Printing Silk-Based Bioresorbable Piezoelectric Self-Adhesive Holey Structures for *In Vivo* Monitoring on Soft Tissues

**DOI:** 10.1021/acsami.2c04078

**Published:** 2022-04-19

**Authors:** Irene Chiesa, Carmelo De Maria, Maria Rachele Ceccarini, Lorenzo Mussolin, Riccardo Coletta, Antonino Morabito, Rodolfo Tonin, Martino Calamai, Amelia Morrone, Tommaso Beccari, Luca Valentini

**Affiliations:** † Department of Ingegneria dell’Informazione and Research Center E. Piaggio, University of Pisa, Largo Lucio Lazzarino 1, Pisa 56122, Italy; ‡ Department of Pharmaceutical Sciences, University of Perugia, Perugia 06123, Italy; § Department of Physics and Geology, University of Perugia, Perugia 06123, Italy; ∥ Department of Pediatric Surgery, Meyer Children’s Hospital, Viale Pieraccini 24, Firenze 50139, Italy; ⊥ Dipartimento Neuroscienze, Psicologia, Area del Farmaco e della Salute del Bambino Neurofarba, Università degli Studi di Firenze, Viale Pieraccini 6, Firenze 50121, Italy; # Molecular and Cell Biology Laboratory, Paediatric Neurology Unit and Laboratories, Neuroscience Department, Meyer Children’s Hospital, Firenze 50121, Italy; ¶ European Laboratory for Non-linear Spectroscopy (LENS), University of Florence, Sesto Fiorentino 50019, Italy; ∇ National Institute of Optics-National Research Council (CNR-INO), Sesto Fiorentino 50019, Italy; ○ Civil and Environmental Engineering Department, University of Perugia, Strada di Pentima 4, Terni 05100, Italy; ⧫ Italian Consortium for Science and Technology of Materials (INSTM), Via Giusti 9, Firenze 50121, Italy

**Keywords:** regenerated silk, graphene, tannins, 3D printing, finite element models, self-adhesive piezoelectric 3D printed sensors

## Abstract

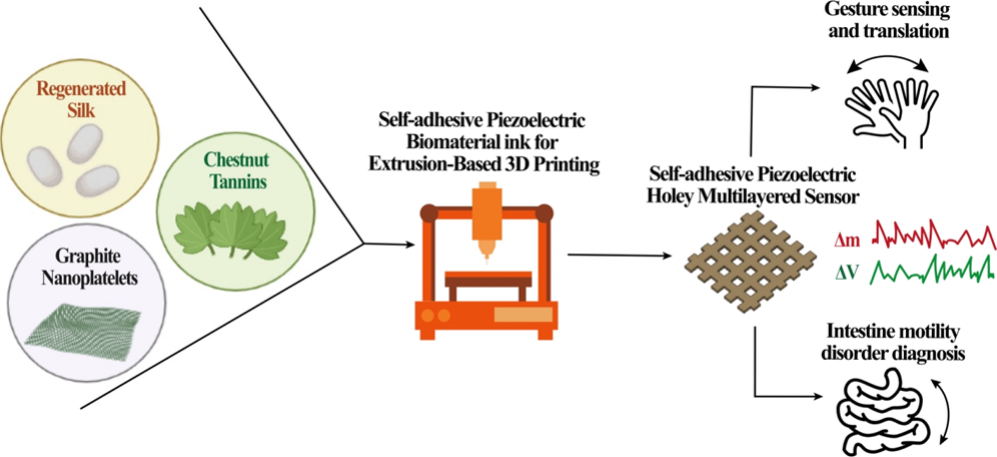

Flexible and biocompatible adhesives with sensing capabilities can be integrated onto human body and organ surfaces, characterized by complex geometries, thus having the potential to sense their physiological stimuli offering monitoring and diagnosis of a wide spectrum of diseases. The challenges in this innovative field are the following: (i) the coupling method between the smart adhesive and the soft human substrates, (ii) the bioresorbable behavior of the material, and (iii) the electrical exchange with the substrate. Here, we introduce a multifunctional composite by mixing silk fibroin, featuring piezoelectric properties, with a soluble plant-derived polyphenol (*i.e.*, chestnut tannin) modified with graphene nanoplatelets. This material behaves as a glue on different substrates and gives rise to high elongation at break, conformability, and adhesive performances to gastrointestinal tissues in a rat model and favors the printability *via* extrusion-based 3D printing. Exploiting these properties, we designed a bioresorbable 3D printed flexible and self-adhesive piezoelectric device that senses the motility once applied onto a phantom intestine and the hand gesture by signal translation. Experimental results also include the biocompatibility study using gastrointestinal cells. These findings could have applicability in animal model studies, and, thanks to the bioresorbable behavior of the materials, such an adhesive device could be used for monitoring the motility of the gastrointestinal tract and for the diagnosis of motility disorders.

## 1 Introduction

Regenerated silk (RS), due to its biocompatibility, biodegradation, and piezoelectric properties,^1−3^ has been used in different studies as interface material between living tissues and electronic devices for cardiac sensors, brain electrodes, and electronic skin.^4−7^ Devices based on active piezoelectric RS with easy and reproducible fabrication techniques, fast application, and bioresorbable properties offer a multifunctional platform for sensing.

Tannins are biocompatible molecules produced by superior plants as a protection against oxidative processes and against biologic attacks from fungi and bacteria.^8^ It is noteworthy that tannins interact with proteins, producing stable complexes hindering the secondary bonding between the protein chains (intra- and inter-molecular) and establishing a stable complex through non-covalent bonds.^9^ Based on our previous work, we observed that tannin plays a crucial role in generating a solid material from a viscous one based only on RS, graphene, and calcium chloride (CaCl_2_).^10^ Graphene, on the other hand, due to its electrical^11^ and mechanical properties,^12^ has therefore been utilized for biosensor fabrication. Here, we have combined 3D printing technologies (*i.e.*, the fabrication of 3D objects layer by layer starting from a digital model) with the self-adhesive properties of these materials on different substrates along with their sensing properties to fabricate smart adhesive devices for *in vivo* monitoring.

Flexible and conformable electronics takes advantage from 3D printing, due to the possibility to fabricate multi-material complex objects with reproducibility and accuracy.^13−15^

Moreover, 3D printing allows to precisely define and control the microstructure of the manufactured part in an easy way, playing on the internal geometry of the printed object during the slicing process. Thus, the technology allows to tune the mechanical behavior of the structure with an unprecedented easiness in order to mimic the mechanical response of the target tissue.^16,17^ In this scenario, finite element (FE) simulations could be extremely useful to study the stress, strain, and the global displacement of the 3D printed structure according to the variation of the infill density and pattern and to compare this response with the target tissue.

Silk-based materials mimic the conduction mechanism of organs by conducting electricity *via* ions trapped inside^18−21^ and generate electrical signals between metal electrodes through an intimate communication link between the underlying soft substrate and unanimated 3D sensor. Targeting this field of applications, 3D silk-based devices require an appropriate level of ionic conductivity and bioresorbability with rates that match its use in biomedical implants.^22,23^

In this work, we show the development of a functional 3D printed smart adhesive device, which mixes and matches the requirements reported above. The device combines the synergistic effect of the addition of graphene and tannin to RS that supports fast self-adhesion on different substrates and piezoelectric properties. The 3D printability of the graphene–RS/tannin (G-RS/T) solutions to fabricate multilayer grids was investigated and proved. FE models were implemented to study the mechanical behavior of the 3D printed grids. Moreover, the 3D printing methodology exploited in this study allowed the easy transfer of the device from the printing substrate (*i.e.*, a water-soluble film) to complex geometries without application of external forces or tools, thereby reducing the risk of damages due to its handling, or the use of not human-compatible methods, such as heat.

An important part of this work is a gallery of proofs of concept with the 3D printed piezoelectric and adhesive device interfaced to soft surfaces, that mimic vital organs in the context of stimulation and electrical measurement. Examples include the harvesting of the mechanical energy from the gastrointestinal tract for the diagnosis of motility disorders to a smart glove for hand gesture sensing and sign translation. These results show the broad spectrum of possibilities enabled by this new class of adhesive material.

## 2 Materials and Methods

### 2.1 Materials

Silk cocoons were supplied by a local farm (Fimo srl, Milano, Italy). Sodium hydrogen carbonate (NaHCO_3_), CaCl_2_, and formic acid (FA) were supplied by Merck (Darmstadt, Germany). Graphite nanoplatelets C777 [GNPs, carbon content > 65%, average flake thickness ∼ 20 nm, layer number ∼ 41, average particle (lateral) size: 16 μm] were supplied by Nanesa (Arezzo, Italy). Commercial chestnut tannin extract “Saviotan A” was kindly supplied by Saviolife (Viadana, Italy).

### 2.2 Synthesis of G-RS/T Films

*Bombyx mori* silk cocoons (10 g) were boiled for 30 min in 200 mL of water containing 5 g of NaHCO_3_. The extracted fibers were washed two times with water and dried at room temperature under a chemical hood in laminar flow. Subsequently, the fibroin fibers were dissolved in 5 mL of FA containing an amount of CaCl_2_ equal to 60:40 with respect to the weight of the dried fibroin fibers (*i.e.*, 0.65 g) at 30 °C for 1 h. Afterward, 1, 2, 5, and 10 wt % of tannin, with respect to the silk content, was added to the solution, leaving the dissolution to proceed at 50 °C. Finally, 1 wt % of GNPs, with respect to the silk amount, were added to the solutions and sonicated for 30 min at room temperature. The rationale behind utilizing 1 wt % GNPs was the current–voltage characterization of RS added with 0.5 and 2 wt % and observing a maximum of the electrical current for that at 1 wt % of GNPS (Figure S1).

Films were then fabricated by drop casting the solutions into Petri dishes with a diameter of 5 cm and left to evaporate at 40 °C for 4 h. Hereinafter, RS/T is used for samples obtained by adding only tannin and G-RS/T for those obtained by adding also GNPs. For comparison purposes, we also prepared films of pure RS and RS modified with graphene (*i.e.*, G-RS).

### 2.3 Material and Film Characterization

To evaluate the chemical interactions in the composites and structural changes of the silk fibroin, Fourier-transform infrared spectroscopy (FTIR) analysis was conducted on the films using a PerkinElmer Spectrum 100 (USA) FTIR spectrometer equipped with attenuated total reflectance. Data were recorded at room temperature between 4000 and 500 cm^–1^ for 64 scans at a resolution of 4 cm^–1^. The spectra in the 1450–1750 cm^–1^ range where deconvoluted using Origin 9 software (OriginPro, version 9.0, OriginLab Corporation, MA, USA) applying the PeakFit routine function with Gaussian-like peaks, to determine secondary structures of silk proteins. For the calculation of the composition of secondary structures of silk fibroin, the peaks related to the C–C and C=O from tannin were eliminated.

The stress–strain curves of the RS, G-RS, RS/T, and G-RS/T films (3 cm × 1.5 cm rectangle-shaped samples, 500 μm mean thickness) were obtained through a tensile testing machine (Lloyd Instr. Ltd. LR30K, Steyning Way West Sussex, UK). The samples were tested at room temperature with a strain rate of 5 mm·min^–1^ using a 50 N load cell. Three samples per formulation were tested.

The adhesive properties were measured with lap shear strength experiments (ASTM F2255) on wood, steel, and latex surfaces using Instron Testing Systems in the tensile mode with a 500 N load cell. Wood, steel, and latex weights with dimensions of 10 mm × 40 mm were adhered with RS, RS/T, or G-RS/T adhesive and pressed gently. The adhesion strength was calculated as the maximum load divided by the bond area.

Bioresorbability was evaluated by soaking the prepared samples in phosphate buffered saline (PBS) (pH 7.4 at 37 °C) for 21 days, washing the specimens in distilled water to remove residual PBS, and measuring the dried weights. The percentage of the remaining material was expressed as the ratio of the dried weight to the original one.

The piezoelectric effect was measured using a Keysight DAQ970A data acquisition system equipped with a DAQM901A 20 channel multiplexer configured for measuring DC voltage with 100 ms sampling time.

### 2.4 Cytotoxicity Assay

*Caco2*, a human colon adenocarcinoma cell line, was purchased directly from the ATCC (Manassas, VA). Caco-2 cells were maintained at 37 °C in Dulbecco’s modified Eagle’s medium (DMEM), containing 10% fetal bovine serum (FBS), 1% nonessential amino acids (NEAAs), 2 mM l-glutamine, and antibiotics (100 U/mL penicillin and 100 μg/mL streptomycin) in an atmosphere of 5% CO_2_ and 90% relative humidity. Medium was changed every other day of culture, and cells were passaged at 80–90% confluence at a split ratio of 1:3 as recommended. Cells were passaged five times in each medium before use in experimentation.

RS, RS/T, and G-RS/T cytotoxicity was evaluated using the 3-(4,5-dimethylthiazol-2-yl)-2,5-diphenyltetrazolium bromide (MTT) method as previously described.^22^ Each stock solution was prepared incubating the silk-based solution (1 mg/mL) with complete medium (DMEM) for 1 h at 37 °C. Eight scalar dilutions from 7.8 μg/mL to 1.0 mg/mL were tested after 24 and 48 h of incubation. Optical density values were measured spectrophotometrically at 570 nm (Eliza MAT 2000, DRG Instruments GmbH, Marburg, Germany). Each experiment was performed in triplicate, and cell viability was expressed as a relative percentage, as previously described.^24^

### 2.5 Adhesion Test in an *In Vivo* Gastrointestinal Study in Rat Model

Institutional approval for the *in vivo* gastrointestinal study in the rat model was granted from Ministry of Health authorization no. 226/2020-PR. Albino Wistar rats with a weight between 300 and 350 g were selected for the animal model. The animals were housed in social groups of two–three subjects in static cages with environmental enrichment (material for nest/gnawing, shelters) and had diet and water available ad libitum. Before surgery, rats were premedicated with carprofen 5 mg/kg and buprenorphine 0.05 mg/kg subcutaneously (SC) for analgesia. After 30 min, the rats undertook to general anesthesia using a mixture of oxygen and 5% isoflurane in the induction chamber. The achievement of the adequate depth of anesthesia was monitored with light noxious stimuli, such as tail pinch or toe pinch. To prevent post-operative infection, a single shot of enrofloxacin 10 mg/kg SC was administered before surgical incision. After laparotomy, a colonic segment of 10 cm was identified and transected to create an intestinal anastomosis by an interrupted 6/0 PDS suture. In half of the animal, RS-based films were applied on the serosal surface circumferentially. The bowel was then replaced in the abdominal cavity, and the abdomen was closed in a 4/0 Vicryl Plus suture. At post-operative day 10, the animals were sacrificed, and the 10 cm intestinal segment with the anastomosis in the middle was retrieved.

Burst strength was used as a surrogate marker to measure intestinal anastomotic integrity. Each intestinal anastomotic site was isolated and ligated proximally and distally using 2 square knots of umbilical tape in preparation for burst strength testing. A catheter was then inserted into the proximal end of the isolated segment and secured with a surgical suture to create a watertight seal. Each intestinal segment was tested separately. A customized system consisting of a peristaltic pump was used, together with a digital manometer, to measure and record the bursting pressure (*i.e.*, maximum pressure reached followed by a sharp loss in pressure) of the anastomosis; both the intact intestine and sutured intestine with the anastomosis were treated with the RS-based films.

### 2.6 Pre-printing Material Characterization

Rheological measurements and contact angle measurements on different substrates were performed as essential analysis to define the printability window of the desired materials [*i.e.*, (i) G-RS; (ii) G-RS/T1; (iii) G-RS/T10; (iv) RS; (v) RS/T1%; and (vi) RS/T10%] for fabricating multilayer grids through the extrusion-based 3D printing process.^13,25−27^

Rheological measurements of the selected materials were performed at room temperature using a HAAKE RheoStress 6000 rheometer (Thermo Scientific) equipped with a cone-plate (1° angle) measuring tool. To assess the viscosity behavior during the extrusion process, the steady-state flow curve in the control shear rate mode was performed in the shear rate range of 10–2000 1/s.^27^ To evaluate the eventual presence of a yield stress, a tangent cross-over method was used.^25^ An amplitude sweep was performed in the control stress mode (shear stress range: 0.01–500 Pa) at 1 Hz.

Contact angle measurements of the selected materials were performed at room temperature using an optical tensiometer (Attension Theta Lite, Biolin Scientific). A sessile drop analysis was performed according to the Young–Laplace analysis mode. Laboratory glass slides, that is a commonly used 3D printing substrate, and a water-soluble polymer layer (2 mg/mL Hydrofilm, Lucart, Italy, which is the printing substrate used in this work, see the next section)^28^ were tested.

### 2.7 3D Printing Process

**Figure 1:**
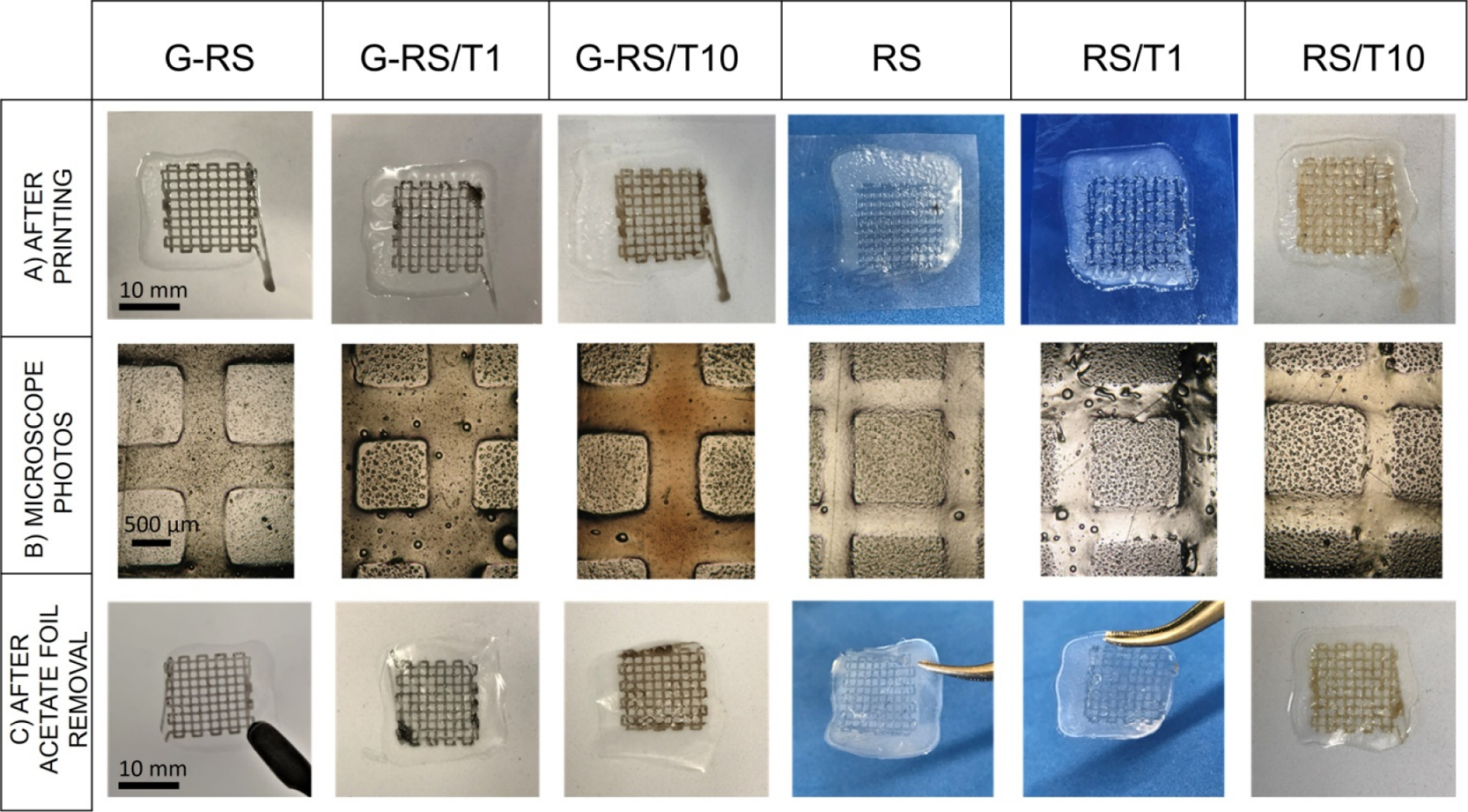
3D printed grids based on RS, tannin, and GNP. (A) Photos taken as soon as the printing stopped. (B) Photos obtained with a brightfield microscope. (C) Photos of the structures after the acetate foil removal.

Multilayered grid structures were 3D printed on a water-soluble polymer layer (2 mg/mL Hydrofilm)^28^ attached to an acetate foil using a custom-made piston-driven extrusion-based 3D printer.^13,22^ The gcode of the structure was generated by using Slic3r starting from a 1.5 cm × 1.5 cm × 200 μm parallelepiped and applying the following printing parameters: infill = 15%; print speed = 5.5 mm·s^–1^; volumetric flow = 0.18 mm^3^·s^–1^; needle diameter = 0.21 mm; and layer height = 50 μm (four layers in total). Six different solutions were used: (i) G-RS; (ii) G-RS/T1; (iii) G-RS/T10; (iv) RS; (v) RS/T1; and (vi) RS/T10 (Figure 1A). After the printing, the structures were dried at room temperature for 24 h to allow any FA residual to evaporate. Then, for each RS solution, images of the grid lines and pores were acquired with a brightfield microscope (Olympus AX70) (Figure 1B), and the width of the lines and pores were measured by using ImageJ. Finally, the RS-based grids, still attached to the Hydrofilm, were removed from the acetate foil (Figure 1C) and stored at room temperature until further use.

### 2.8 FE Modeling for Tuning the Mechanical Behavior

**Figure 2:**
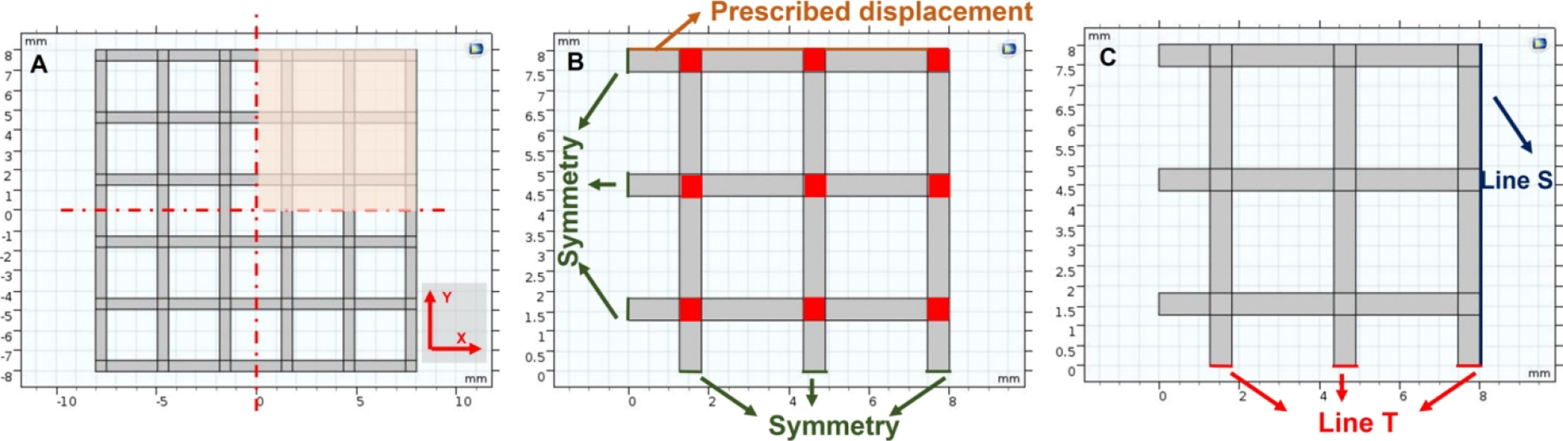
Design of the implemented FE models when the 15% infill density is used. (A) Schematic of the complete grid. (B) Boundary conditions set in all the simulations. Red squares indicate the domains with a 100 μm out-of-plane thickness. (C) Boundaries of interest along which the derived variables were evaluated.

FE models were implemented in COMSOL Multiphysics (Comsol Inc., 5.3) to study the effect of the grid micropattern on the mechanical behavior of the structure, obtained by varying the infill density printing parameter (*i.e.*, 15, 30, 50, 75, and 100%) and the material (*i.e.*, RS, G-RS, G-RS/T1, G-RS/T10, RS, RS/T1, and RS/T10). The infill densities between 15 and 75% mimic a 3D printed structure, whereas the 100% infill density represents a structure fabricated *via* drop casting. In all simulations, the solid mechanics application mode under static conditions was used, with the 2D plane stress analysis.^16^ To model the different infill densities, five microstructures were used for each material with equal bulk dimensions (*i.e.*, 16 mm × 16 mm) and line thickness (*i.e.*, 550 μm), while the dimensions and numbers of pores varied according to the infill density. For simplicity, squared grids and pores were designed (Figure 2A,B). The out-of-plane thickness was set equal to 50 μm, but where the grid lines with different orientation overlapped (Figure 2B, red squares), the thickness was set at 100 μm. All domains were set as a linear elastic material according to Table 1. Then, a prescribed exploratory displacement along the *y*-axis equal to 1 mm was imposed as the boundary condition (Figure 2B, orange). A symmetry boundary condition was added along two sides of the structure, thus allowing to simulate a quarter of the whole structure (Figure 2A,B, green). A triangular mesh, controlled by the physics, was used in all the simulations. Mesh statistics are shown in Table S1. As a control, a 100% infill model mimicking a soft tissue was simulated, and its subdomain settings are reported in Table 1. Then, for all the implemented models, we evaluated the following: (i) the displacement along the *X*-axis on boundary *S* (Figure 2C, blue); (ii) the reaction forces along the *Y*-axis on boundary *T* (Figure 2C, red), from which generalized elastic spring constant *k* was calculated as in eq 1; and (iii) the maximum von Mises stress.
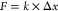
1

**Table 1:** Subdomain Settings Set in the FE Model for All the Tested RS-Based Solutions and for the Soft Tissue

material	elastic modulus [MPa]	density [kg/m^3^]	Poisson ratio
RS	0.17	1400	0.3
G-RS	5.1	840	0.3
RS/T1	0.5	1410	0.37
G-RS/T1	2.7	840	0.4
RS/T10	0.27	1450	0.38
G-RS/T10	0.94	850	0.45
soft tissue	0.1	1000	0.49

### 2.9 Biocompatibility

In order to evaluate the biocompatibility of RS-based grids, human skin fibroblasts obtained from primary cultures were seeded and grown on RS-based grids under static culture conditions with DMEM with FBS (10%) and 2 mM l-glutamine and antibiotics (100 U/mL penicillin, 100 μg/mL streptomycin) for 15 days in a 6-well plate. DMEM medium was changed every 3–4 days. Fibroblast growth and confluence were evaluated daily by using an inverted microscope. Grids were sterilized by 1 h exposure to the ultraviolet light (UV-C) in a biosafety level 2 cabinet. Anonymous skin fibroblast cell lines were used in compliance with the ethical recommendations issued and the International Declaration on Human Genetic Data of 2003. Samples incubated with cells were washed with PBS and fixed using 4% paraformaldehyde. After rinsing with PBS and blocking with 4% bovine serum albumin in PBS, the samples were fluorescently labeled after simultaneous incubation for 15 min with Hoechst 33342 (Thermo Fisher) at a concentration of 1 μg/mL and with wheat germ agglutinin 568 (WGA568, Thermo Fisher), at 5 μg/mL. Imaging was performed with a confocal microscope (Nikon Eclipse TE300), equipped with the Nikon C2 scanning head Coherent CUBE (diode 405 nm) and Coherent Sapphire (Sapphire 561 nm) lasers. Emission filters for imaging were 452/45 and 595/60 nm.

### 2.10 Proofs of Concept

We used a commercial platinum-catalyzed silicone Ecoflex 00-10 to build a phantom intestine (tensile strength 120 psi, 100% modulus 8 psi, elongation at break 800%, shore hardness 00–10; inner diameter 30.0 ± 0.1 mm and outer diameter 32.0 ± 0.1 mm). The 3D printed grids, still attached to the Hydrofilm, were placed onto the substrate (*i.e.*, on the external side of the intestine phantom) and washed with distillated water until the Hydrofilm layer dissolved, thus allowing the grids to be transferred and self-adhere to the surface of the phantom intestine. Then, the grids were left to evaporate and then connected between two adhesive Cu electrodes. A digital manometer was connected in series to a pump recording the pressure of the water flowing inside. While the water flows inside the phantom intestine, the open-circuit voltage values were monitored using a computer-controlled Keithley 4200 Source Meter Unit (Tektronix UK Ltd., The Capitol Building, Oldbury, UK). The dynamic piezoelectric output of the samples was measured by using the finger imparting method reported elsewhere.^29^ As potential implantable biosensors, we investigated their sensing characteristics every 7 days by immersing the grids in PBS (*i.e.*, 37 °C; pH 7.4). Moreover, to test the mechanical properties of the grids under cyclic conditions, a clear latex balloon was used to simulate the stand-in for the intestine. The grid was adhered to the balloon with the procedure reported above (*i.e.*, wet conditions). The balloon was cyclically inflated with air gas at a constant pressure equal to 40 kPa for 15 s three times (see Video S2).

In order to sense the hand gesture, the 3D printed grid was adhered on the top of a glove as described above. Then, the smart grid was placed between two Cu electrodes and connected to a computer-controlled Keithley 4200 Source Meter Unit (Tektronix UK Ltd., The Capitol Building, Oldbury, UK). Any change in the grid length, due to the hand movement, results in a change in its voltage output, proportional to the hand strain. The data were collected by mapping different hand movements.

### 2.11 Statistical Analysis

GraphPad Prism 9.2.0.332 (GraphPad software, San Diego, CA, USA) was used to assess the statistical significance of all comparison studies in this work. In the statistical analysis for comparison between multiple groups, a two-way analysis of variance (ANOVA) with Tukey’s post-hoc analysis (multiple comparison) was conducted, while a *T*-test was used when comparing two groups. In both cases, the significance threshold of *P < 0.05, **P ≤ 0.01, and ***P ≤ 0.001 was chosen. For cytotoxicity data, one-way ANOVA analysis was performed with the significance threshold at *p < 0.01, **p < 0.001, and ***p < 0.0001.

## 3 Results and Discussion

### 3.1 Material and Structure Characterization

**Figure 3:**
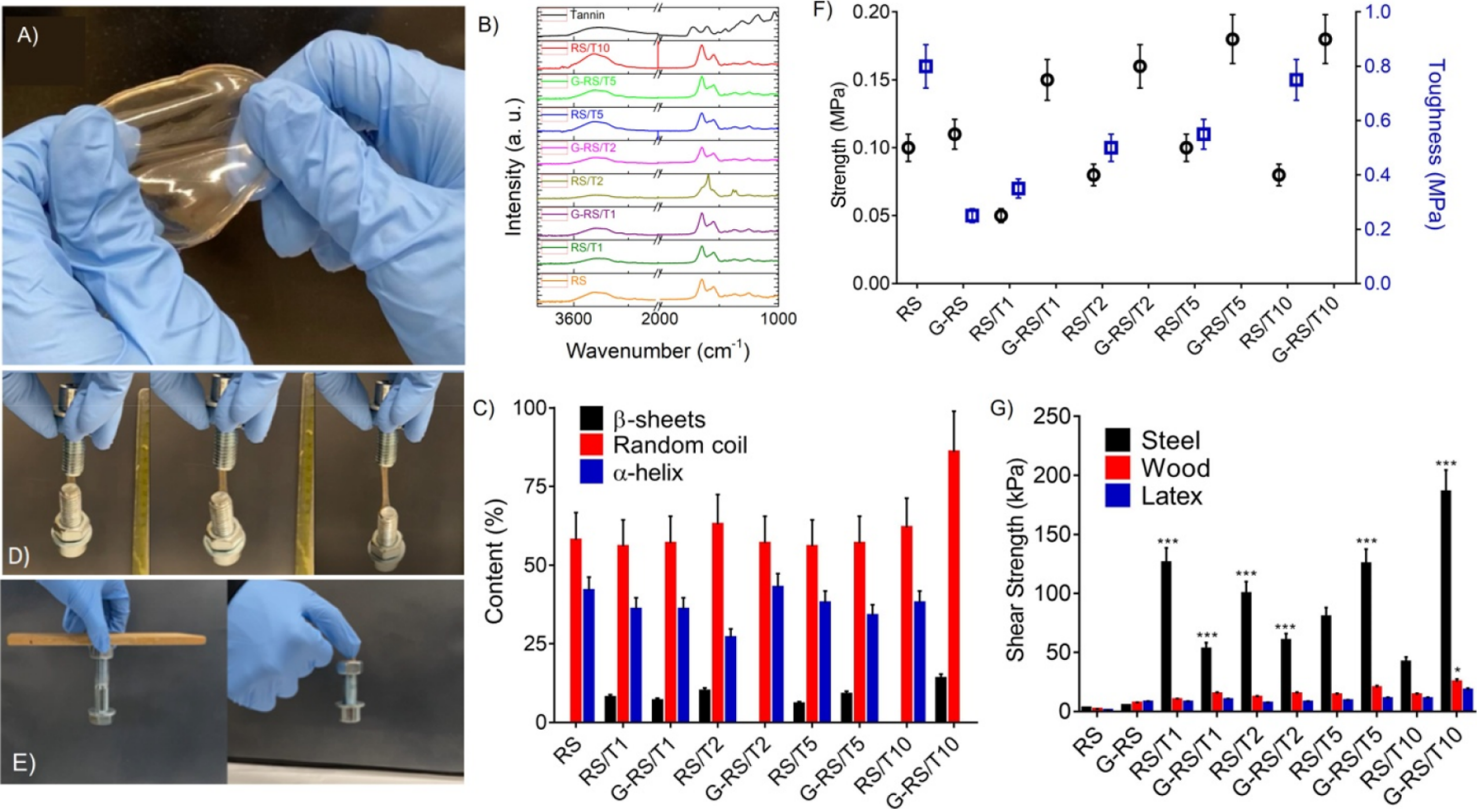
(A) Photograph of the RS/T10 composite. (B) FTIR spectra of neat RS, RS/T, and G-RS/T composites. (C) Structure composition of the prepared specimens. (D) Photographs showing the cohesive adhesion on stainless steel of the RS/T10 composite and tensile strength. (E) Adhesion of RS/T on wood (left) and latex (right). (F) Toughness of the prepared specimens calculated from the engineering stress–strain curves (Figure S2). (G) Shear strength measurements of the prepared specimens.

By combining RS and tannin, the resulting material was reminiscent of something in between a solid and a hydrogel with a gum-like behavior (Figure 3A). The FTIR spectra of RS/T samples (Figure 3B) show the signature peaks associated with RS, such as amide I (1642 cm^–1^), amide II (1515 cm^–1^), and amide III (1235 cm^–1^) and tannin, such as C–O–C stretching (1308 cm^–1^), in accordance with previous reports.^30^ The hydrogen bonds forming between tannin and RS resulted in a wavenumber shift to 3323 cm^–1^ of the tannin-associated hydroxyl group (OH) from 3400 cm^–1^ and the amide (NH)-associated peak of RS centered at 3290 cm^–1^. As shown in Figure 3C, the addition of graphene gives rise to the appearance of β-structures. According to previous studies,^31,32^ the interfacing of proteins with 2D materials can induce changes from random coils to crystalline β-structures through surface-induced polypeptide chain folding. A two-way ANOVA test conducted on each of secondary structures among the different samples revealed no statistical differences (Figure 3C); that is, none of the secondary structures were correlated with the synergistic effect of addition of tannin and GNPs.

Soft materials based on ion inclusions typically utilize bridging between silk fibroin chains and ions to obtain stretchable materials. In our case, Ca^2+^ ions in RS capture water molecules from the atmosphere, and these, as plasticizers, result in much softer and stretchable films (Figure 3D). The mechanical properties reported in Figure 3D also illustrate the synergistic effect of the addition of both tannin and graphene for the RS composite. As a result, the toughness of the G-RS/T samples increased, with a sort of saturation effect on the mechanical strength observed for the G-RS/T10 sample. The mechanical properties of the RS added with different tannin concentrations indicate instead a decrease in the tensile strength; these findings can be rationalized by assuming that the molecules of tannin hinder the secondary bonding between the protein chains (intra- and inter-molecular), establishing a stable complex through non-covalent bonds which facilitates the sliding of the molecules, so the tensile strength reduces.^33^

The oxidized polyphenol groups of tannins enable adhesion through catecholamine-like chemistry mimicking the mussel adhesion mechanism.^34,35^ In Figure 3E, we show a simple gravity-based test wherein a RS/T film was sandwiched between a weight and various materials, including wood and latex. The images demonstrate the adhesive property on both surfaces. We then quantified the adhesive strengths (Figure 3G) of RS/T and G-RS/T films to steel, wood, and latex interfaces as described above. From these results, it is evident that the adhesive strength increased as GNPs were added. These results are in agreement with those of a previous study that^36^ shows how graphene improves the cohesion strength of the graphene-based adhesives.^36^

**Figure 4:**
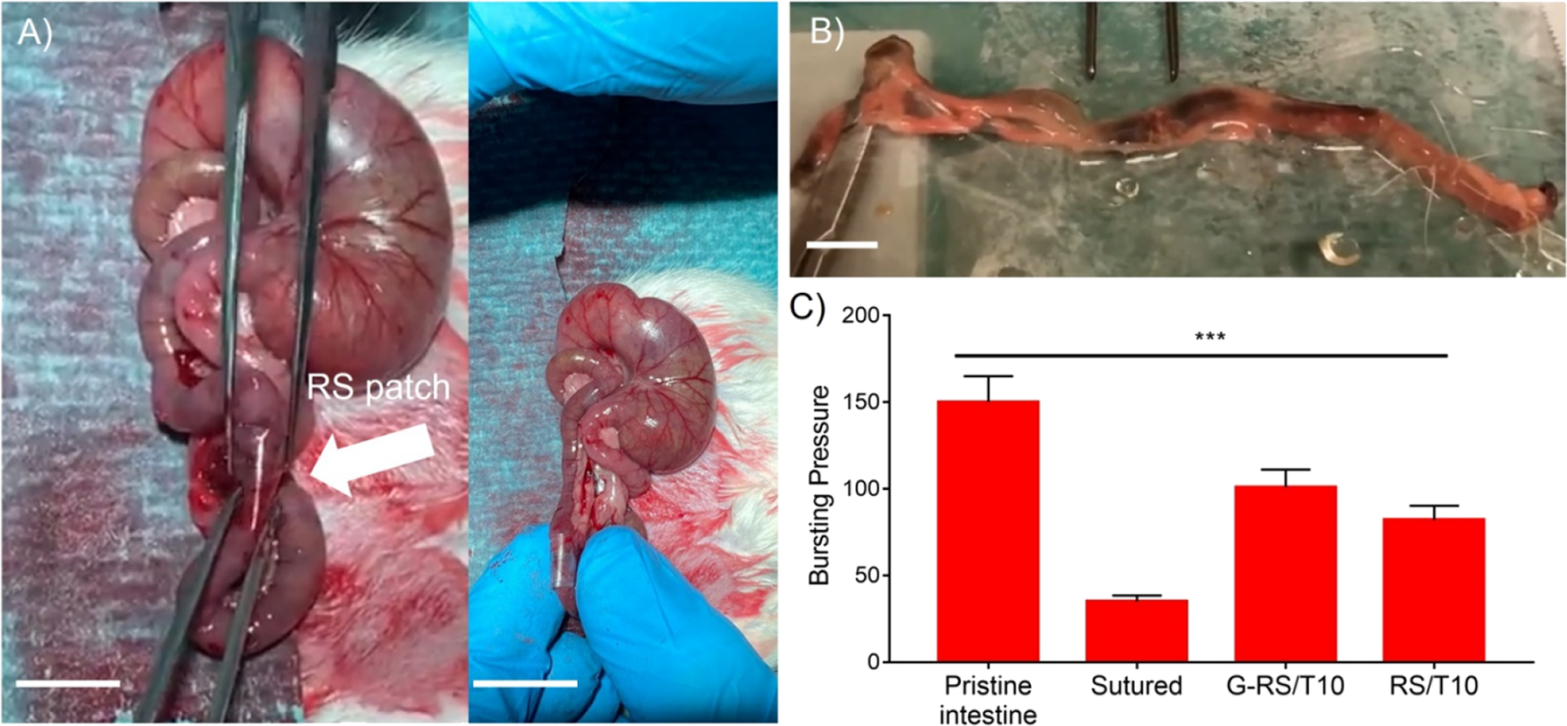
Adhesion performance of the gastrointestinal patch; (A) snapshots of *in vivo* experiments on the rat gastrointestinal segment with RS used as an anastomotic sealer. (B) Burst pressure test of the *ex vivo* rat intestinal anastomosis made by RS-based patches (see Video S1) and (C) results. The scale bars indicate 1 cm.

The coupling method between the smart adhesive and the soft human substrates is a major challenge for sensing adhesives; we further select gastrointestinal rat tissue to monitor the adhesive performance of the RS-based films as patches. The purpose of this experiment is to test the wet adhesion ability necessary for *in vivo* applications. We then evaluated sealing of the gastrointestinal rat by applying the RS patch to the external and wet wall of a rat colon. Therefore, we created an *in vivo* model where the RS patch was used as a sealant for the murine intestinal anastomosis (Figure 4A). After anesthesia, the Wistar rat abdomen was approached and the colon identified. The identified intestinal segment was isolated and transected, and an intestinal anastomosis was performed using an interrupted 7/0 PDS II (polydioxanone) suture. The RS patch was carefully attached circumferentially in half of the rats, and the bowel was replaced in the abdomen. At post-operative day 10, the rats were sacrificed and the intestinal segment with the anastomosis retrieved for further investigation. To test whether the RS patch increased the strength of the anastomosis, the identified segment was resected 5 cm above and 5 cm below the anastomosis, creating a tabularized specimen. After retrieval of the intestinal segment with the anastomosis, the RS patch was completely degraded (Figure 4B). Non-serosal hypertrophy was identified macroscopically, and no intestinal adhesion of the RS patch with other intestinal segments was noted. The strength of the anastomosis was finally tested by bursting the specimen with high pressure. Burst strength appeared to correlate with the presence of the RS patch (Figure 4C and Video S1).

**Figure 5:**
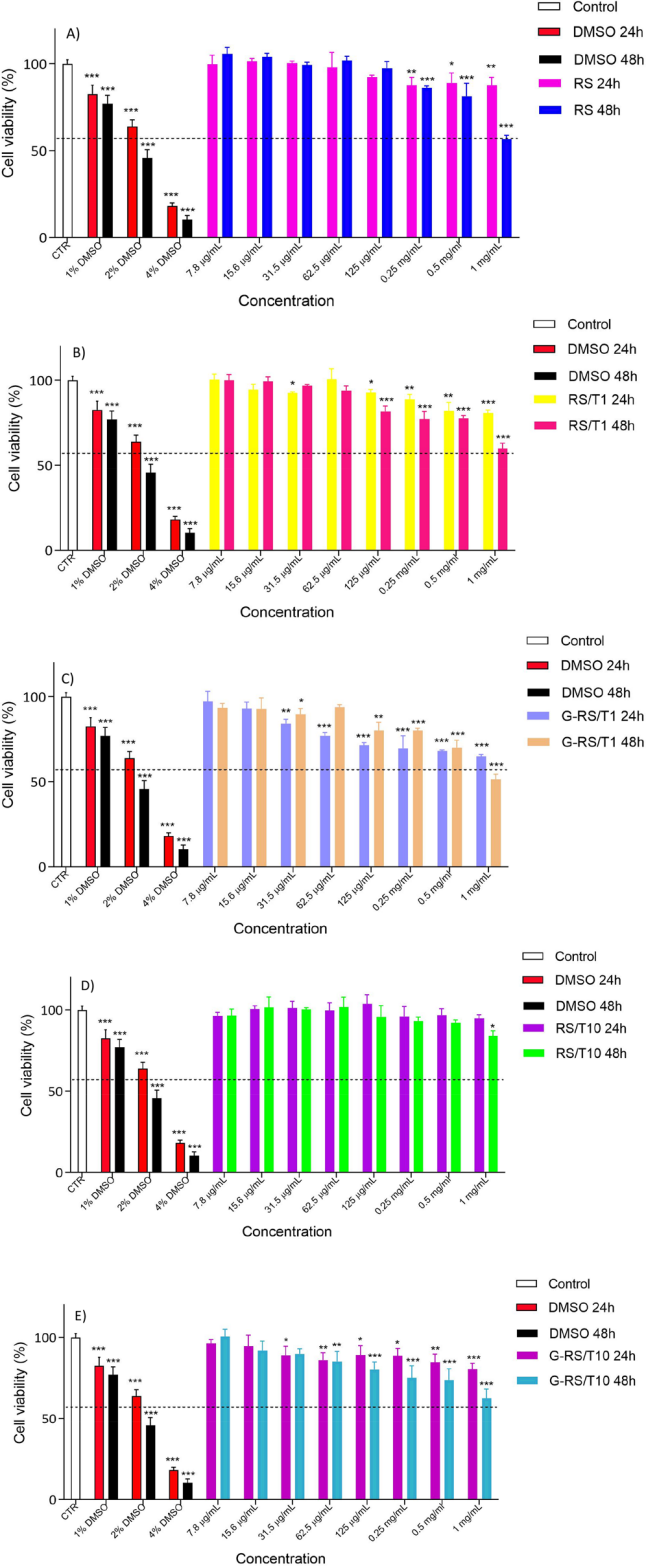
Viability measured *in vitro* on Caco2 cells for (A) RS, (B) RS/T1, (C) G-RS/T1, (D) RS/T10, and (E) G-RS/T10 compounds. Eight scalar concentrations were tested after 24 and 48 h of treatment. Untreated cells (CTR) were set at 100%. The percentage of viable cells with respect to CTR was reported as the mean standard deviation of three independent experiments, each one conducted in triplicate.

Cytocompatibility tests were performed as described above. Based on the MTT results, it is possible to conclude that the RS-based solutions are completely safe for the Caco2 cells line after 24 h of treatment with cell viability ≥80% (Figure 5). Only sample G-RS/T1 shows a low cytotoxic effect after 24 h of treatment at the highest concentrations assayed (0.25, 0.5, and 1 mg/mL). Moreover, a cytotoxic effect was observed after 48 h in a dose-dependent manner at the highest concentrations assayed, namely 0.5 mg/mL (viability < 80%) and 1 mg/mL (viability < 70%), for all films: RS, RS/T1, and G-RS/T1, in particular for G-RS/T10 but except for RS/T10.

The obtained results clearly demonstrated that the RS/T10 is the most promising solution for *in vivo* application, probably thanks to the presence of tannins and its antioxidant activity. The G-RS/T1 resulted as the worst one and, for this reason, requires more in-depth studies. All the other films have a non-cytotoxic effect for the cells from 7.8 to 250 μg/mL. Hence, cell cytotoxicity analysis proved the good biocompatibility of the devices, and they could be considered suitable for future tissue engineering applications.

### 3.2 Pre-printing Assessments

**Figure 6:**
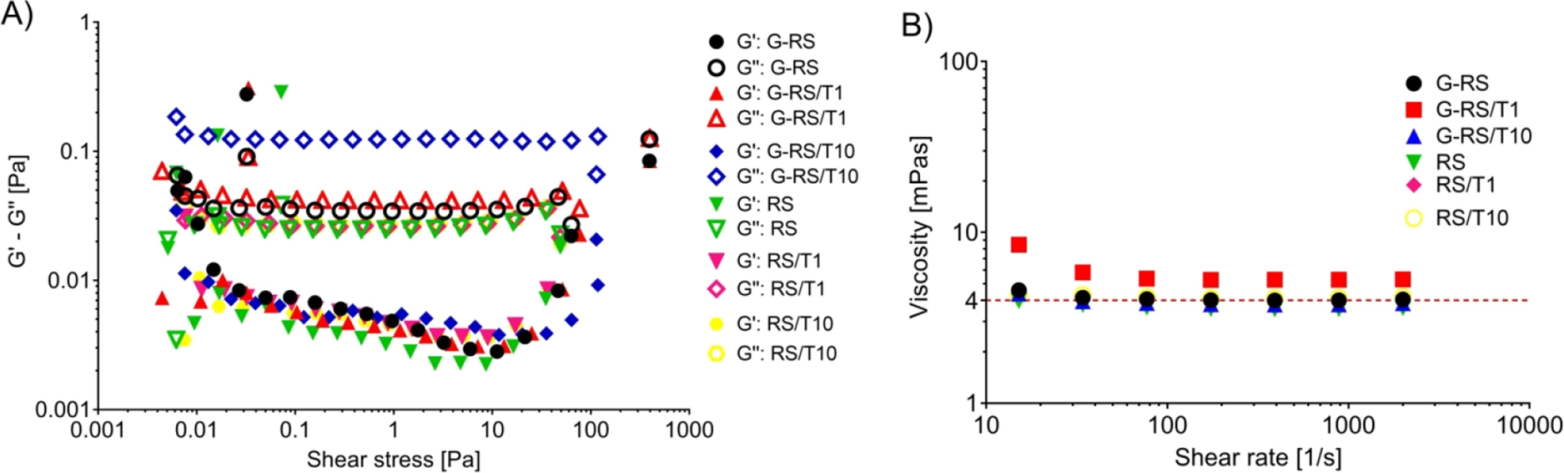
Results from the rheological measurements. In (A), the results from the amplitude sweeps highlight a liquid-like behavior for all the tested solutions. In (B), the results from the flow curve show a Newtonian behavior for all the solutions tested at extrusion-based bioprinting shear rates, with an average viscosity around 4 mPa s (dotted line).

**Figure 7:**
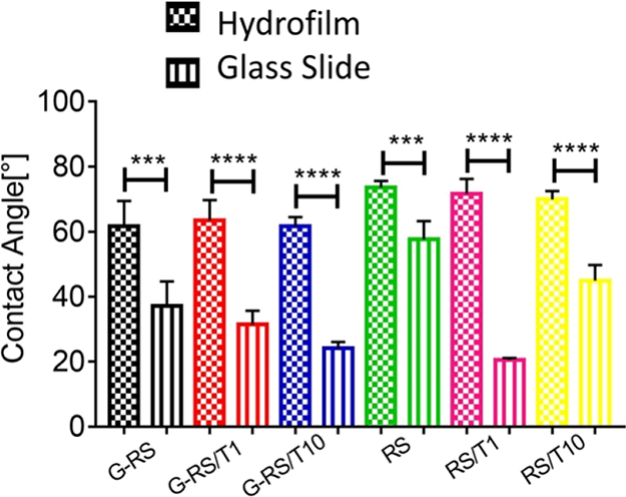
Contact angle measurements [deg] for each solution on the Hydrofilm and glass slides. *T*-Tests highlighted an increase in the contact angle for all solutions when the test is performed on the Hydrofilm, thus showing that the latter is a better printing substrate for the solutions of interest.

To assess the printability of the RS-based solutions in multilayered grids, rheological tests and contact angle measurements were performed. As expected, the oscillatory analysis revealed a liquid-like behavior, with *G*″ bigger than *G*′ for all the tested solutions (Figure 6A). Similarly, the flow curve revealed a Newtonian behavior at the shear rates that characterized extrusion-based bioprinting, that is, larger than 10 1/s.^27^ For all the tested solutions, viscosity is approximately equal to 4 mPa s (Figure 6B). Moreover, no solution showed the presence of a yield stress (data not shown). As clearly stated in the literature,^25,37,38^ those rheological properties do not favor the printability of complex 3D shapes using the selected material. Indeed, a shear thinning behavior with a high yield stress is required to guarantee both the extrudability of the materials and the shape retention of the structure. However, since the aim of this paper is the microfabrication of four-layer grids, the rheological properties should only provide the easiness of extrudability of the materials, that is granted by the low viscosity at a high shear rate. In the context of this work, the surface energy of the printing substrate is the most relevant property. Contact angle measurements reveal the interaction between the printing substrate and the ink. Indeed, higher contact angles (until certain limits, around 90°) favor shape retention of the printed line on the tested substrate, thus allowing a good printing outcome.^27^ As shown in Figure 7, the contact angle on the Hydrofilm is higher than 60° for each tested solution (Table 2), and there is a statistically significant difference between the test performed on the Hydrofilm and on the glass slide for each solution. Thus, the use of the Hydrofilm as the printing substrate not only allows an easy transfer of the printed structure but also will increase its shape fidelity.

**Table 2:** Contact Angle Measurements Expressed as Mean [deg] ± Standard Deviation

	RS	RS/T1	RS/T10
w/ GNP	66.1 ± 7.8	63.5 ± 6.3	61.6 ± 2.8
w/o GNP	73.6 ± 2	73.5 ± 2.6	69.9± 2.5

### 3.3 Analysis of 3D Printed Structure

**Figure 8:**
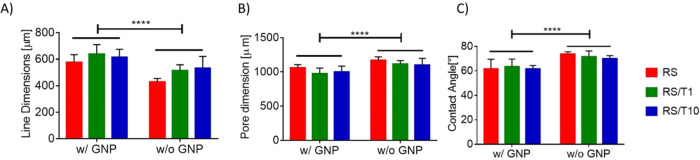
(A) Line and (B) pore dimensions and (C) contact angle for each RS-based solution with and without GNPs. For each measure, two-way ANOVA tests revealed a statistical difference between the samples.

Four-layer grids were 3D printed *via* extrusion-based 3D printing exploiting RS-based solutions that differ in the GNP and tannin contents. Grid lines and pores were measured *via* image analysis, and a mean value for each RS solution was calculated (Table 3). Generally, for all the solutions tested, the line dimension is around 550 μm, thus being more than two times the nozzle diameter (*i.e.*, 210 μm), whereas the pore dimension is around 1000 μm. Statistical analysis revealed that the addition of GNPs and tannin led to an increase in the line dimensions (*p* < 0.0001) (Figure 8A). Accordingly, the addition of GNPs and tannin to the RS solution led to a decrease in the pore dimension (*p* < 0.0001) (Figure 8B). Regarding the GNP addition, the increase in the line dimensions can be associated to the increased wettability of the Hydrofilm by the GNP solution (Figure 8C). Indeed, the RS-based solutions containing GNPs have a lower contact angle if compared with the solution without GNPs (Figure 8C, Table 2), thus leading to higher spreading of the printed line of the GNP-laden solutions. Differently, no statistically significant differences in the contact angles arise when the tannins are added. Thus, we can suppose that the addition of tannin interferes with other aspects of the line formation; for example, we can speculate that the addition of tannin implies a slower evaporation of the FA, thus resulting in an increased line size due to a larger time available for reaching the steady state equilibrium.^27^

**Table 3:** Grid Line and Pore Dimensions for Each RS-Based Solution, Expressed as Mean ± Standard Deviation

	line dimension [μm]			pore dimension [μm]		
	RS	RS/T1	RS/T10	RS	RS/T1	RS/T10
w/ GNP	577 ± 57	638 ± 72	615 ± 60	1059 ± 47	975 ± 80	1001 ± 83
w/o GNP	428 ± 27	514 ± 44	532 ± 89	1170 ± 51	1112 ± 53	1098 ± 100

**Figure 9:**
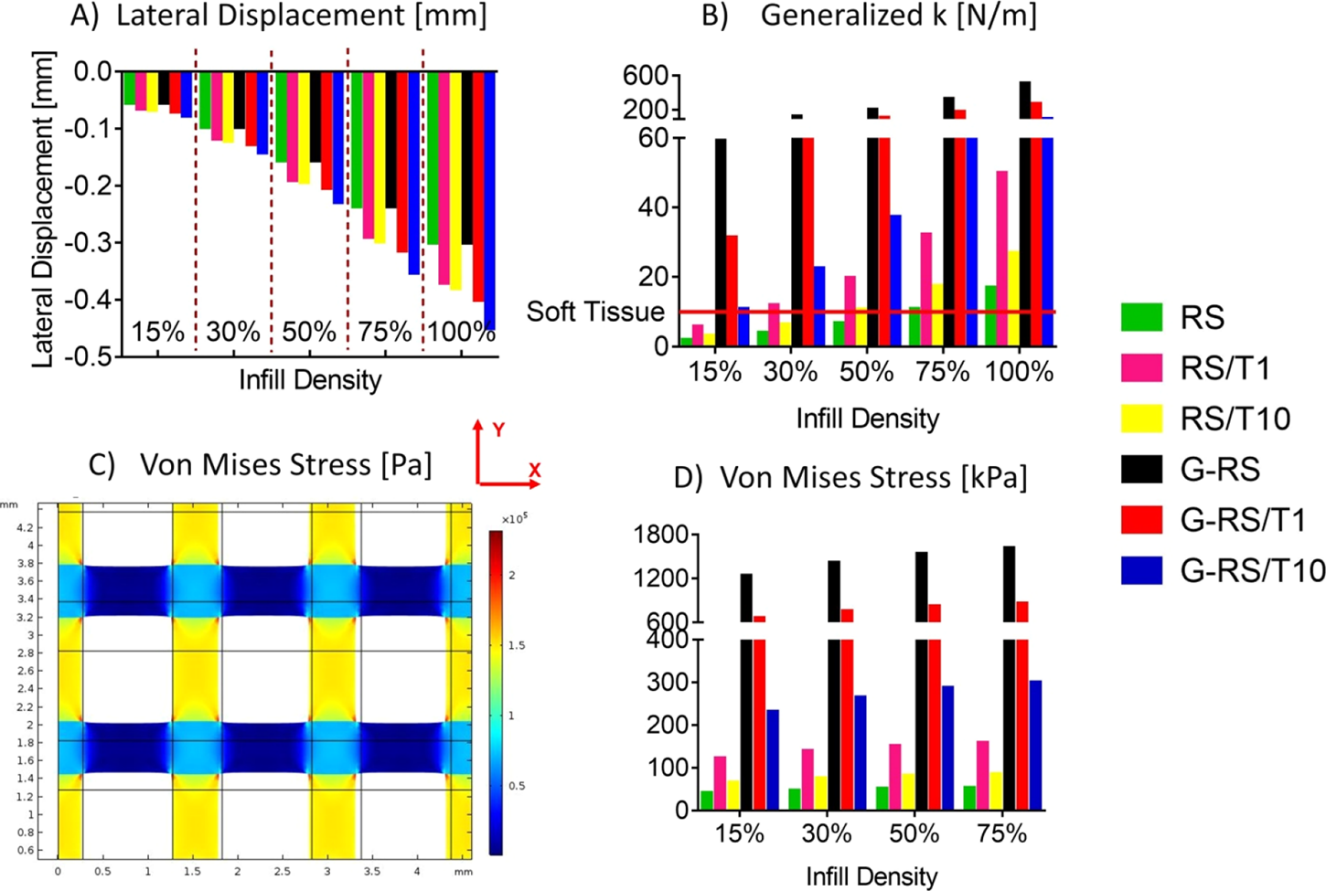
Results from the FE models with varying infill density and material. (A) Lateral displacement along the *X*-axis. (B) Generalized elastic spring constant *k*, the red line indicates the *k* of soft tissues. (C) Zoom-in of the stress tensor along the *Y*-axis of the 30% infill grid that shows the spatial location of the maximum stress value (red spot). (D) von Mises stress.

The peculiar grid geometry of the sensors, obtained through 3D printing, improves their mechanical performance, as showed by the FE models. In more detail, when a holey microstructure is implemented, a reduction of the lateral displacement is achieved (Figure 9A), thus decreasing the unwanted secondary motion of the sensor. Interestingly, this reduction increases as the infill density decreases. In addition, when an infill density lower than 50% is implemented, the generalized *k* is lower than the soft tissue *k* for RS, RS/T1, and RS/T10 (Figure 9B). Therefore, when those solutions are used, the grids will not alter the mechanical behavior of the underlaying soft tissue they are monitoring. Regarding the von Mises stress, its maximum value is in the junctions of the grid parts with different out-of-plan thickness (Figure 9C), due to the line grid overlapping areas that act as rigid nodes, thus reducing the structure motion. Interestingly, the von Mises stress decreases when the infill density decreases (Figure 9D), with a relative difference equal to 14.2, 24.1, and 30.1% when the 15% infill density is compared with the 30, 50, and 75%, respectively. A similar behavior is achieved when rectangular pores are simulated (data not shown). To conclude, FE analysis provides a solid validation of the advantages of 3D printing the grids from a mechanical point of view. In more detail, 3D printing a low-infill micropatterned structure will (i) decrease the unwanted lateral displacement, (ii) decrease the generalized *k*, thus not altering the mechanical response of the underlaying soft tissue; and (iii) decrease the stress of the single grid line.

**Figure 10:**
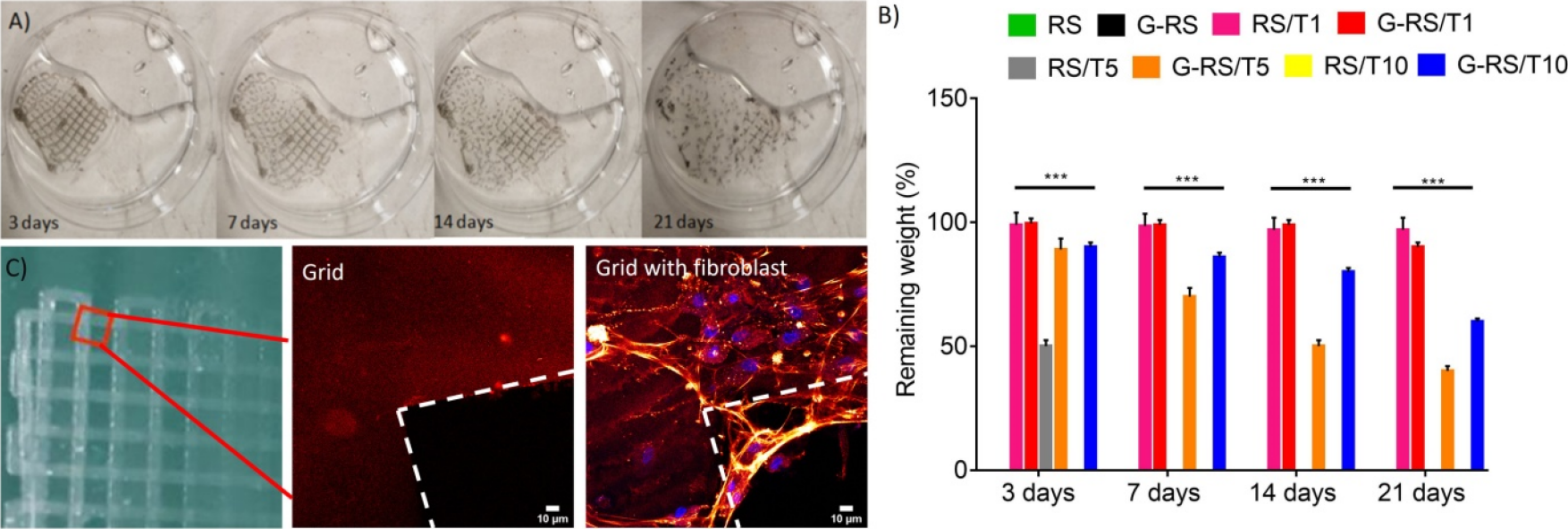
(A) Photographs of the bioresorbable 3D printed grid (*i.e.*, G-RS/T10) in PBS solution (*i.e.*, 37 °C. pH 7.4) and (B) measurements of the rates of dissolution obtained on films. (C) Confocal microscopy images of human fibroblasts seeded on the RS grid. Images were taken at grid intersections. The scale bar indicates 10 mm. The autofluorescence of the grid allows us to highlight the growth of fibroblasts, in which the nuclei (blue) and the plasma membrane (red) are labeled.

Bioresorbability is another important characteristic of all the constituent materials to fabricate piezoelectric devices (see the next section). Figure 10A,B shows photographs of a 3D printed grid and the graph of the remaining weights as a function of time of the prepared specimens after immersion in a PBS solution at body temperature (37 °C) and pH = 7.4. The RS/T samples largely dissolve within 3 weeks, with a difference for the G-RS/T samples that contain a crystalline fraction showing a less pronounced weight loss.

Moreover, the 3D printed RS material is reported to be biocompatible. Indeed, we investigated if cells are able to adhere and spread on the 3D printed grids (Figure 10C). Human skin fibroblasts, one of the most widely studied cells, were used for preliminary assessment of biocompatibility. Figure 10C demonstrates that fibroblasts can proliferate efficiently on the RS deposited on the 3D printed poly(3-hydroxybutyrate-*co*-3-hydroxyvalerate) grid described elsewhere.^13^

### 3.4 Proofs of Concepts

The 3D printed grids were exploited in two proofs of concept as biosensors for gastrointestinal motility and hand gesture. In more detail, the grids were transferred from the printing substrate (*i.e.*, a Hydrofilm layer) to the desired final structure (*i.e.*, an intestine silicone phantom and a glove) by leaning the grids on the desired surface and then dissolving the Hydrofilm with water, therefore without the use of harmful methods, such as heat, or tools or external mechanical forces that could wreck the grids. Then, the strong bio-adhesivity of the grids allows their permanent adhesion to the substrate.

#### 3.4.1 Device for Monitoring the Intestine Motility

**Figure 11:**
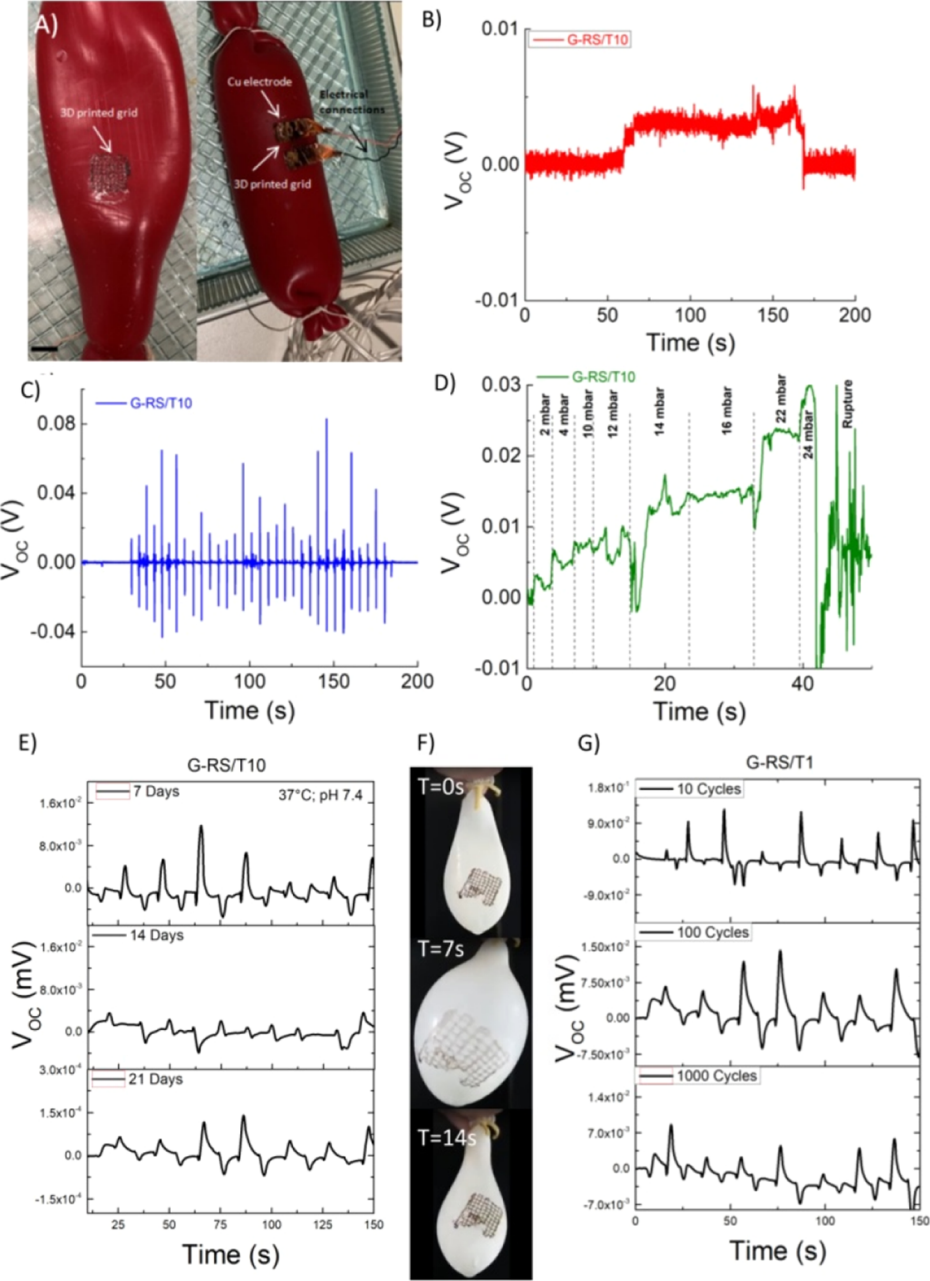
(A) Setup to simulate intestine motility showing 3D printed grids adhered on a phantom intestine. The scale bar indicates 10 mm. (B) Open-circuit voltage output (*V*_OC_) before and after 200 mL of water infusion. (C) Voltage output *vs* finger imparting pressure graph for the prepared specimen. (D) Voltage *vs* time graph during water infusion. (E) *V*_OC_ signals after PBS immersion for 7, 14, and 21 days, respectively. (F) Photographs showing a balloon before and after gas infusion. (G) *V*_OC_ signals recorded on the G-RS/T1 grid after 10, 100, and 1000 cycles of pressure variation, respectively.

We investigated the toughest grid (*i.e.*, G-RS/T10) functionality by performing an experiment with an intestine silicone phantom connected to a flow inlet, a pressure gauge, and a flow outlet to mimic the intestine (Figure 11A). The self-adhesive grids were positioned on the outer wall of the intestine and then water was used to dissolve the Hydrofilm, transferring the grid from the Hydrofilm to the phantom. Then, the grids were electrically connected to a Keysight DAQ970A DMM to collect voltage outputs at a 100 ms sampling rate. Adhesive Cu strips with soldered 24AWG cables were used to connect the grids to the DMM. To simulate fluid ingestion into the intestine, a total volume of 200 mL of water at room temperature was infused with a rate of 2.5 mL s^–1^. The resulting effects before and after water infusion are reported in Figure 11B. When 200 mL of water was infused, the voltage output showed an increase to 5 mV and returned to the base line after the water was removed. The generated voltage increased and decreased when we applied a dynamic mechanical test by applying a constant load every 5 s (Figure 11C). Finally, as shown in Figure 11D, the open circuit voltage generated by the 3D printed grid showed different peaks corresponding to the steps of water infusion. Given that the change in internal pressure generated in the phantom intestine increased with each step, the potential generated follows the mechanical input. In this regard, although the cytotoxicity data indicate that the best composition is RS/T10, we observed a better voltage output for the G-RS/T10 (Figure 11C) sample which has very similar cytotoxicity data to the RS/T10 sample. Following the 3D printed grid bioresorbability shown in Figure 10B, we investigated the sensing characteristics of these grids over time as they dissolved (Figure 11E). This study indicates that the device is sufficiently stable to produce a signal at day 7. At days 14 and 21, the device shows a decrease in the voltage signal to trace the imparting pressure; the functional lifetime follows the bioresorbability data reported in Figure 10B. It should be noticed that the monitoring depends on the requirements specific to each clinical case. Finally, we quantified the device functionality by adhering the grids to a balloon connected to a gas flow inlet, a pressure gauge, and a gas flow outlet to mimic the intestine (Figure 11F,G). The material functionality and its decrease to these cyclic conditions allow us to correlate with the mechanical lifetime under the cyclic test. We perform the test under ambient conditions on the sample (*i.e.*, G-RS/T1) that was found one of the most stable after 21 days in PBS. Our tests support the idea that this material is sufficiently strong for long-term monitoring tests.

#### 3.4.2 Device for Hand Gesture Recognition

**Figure 12:**
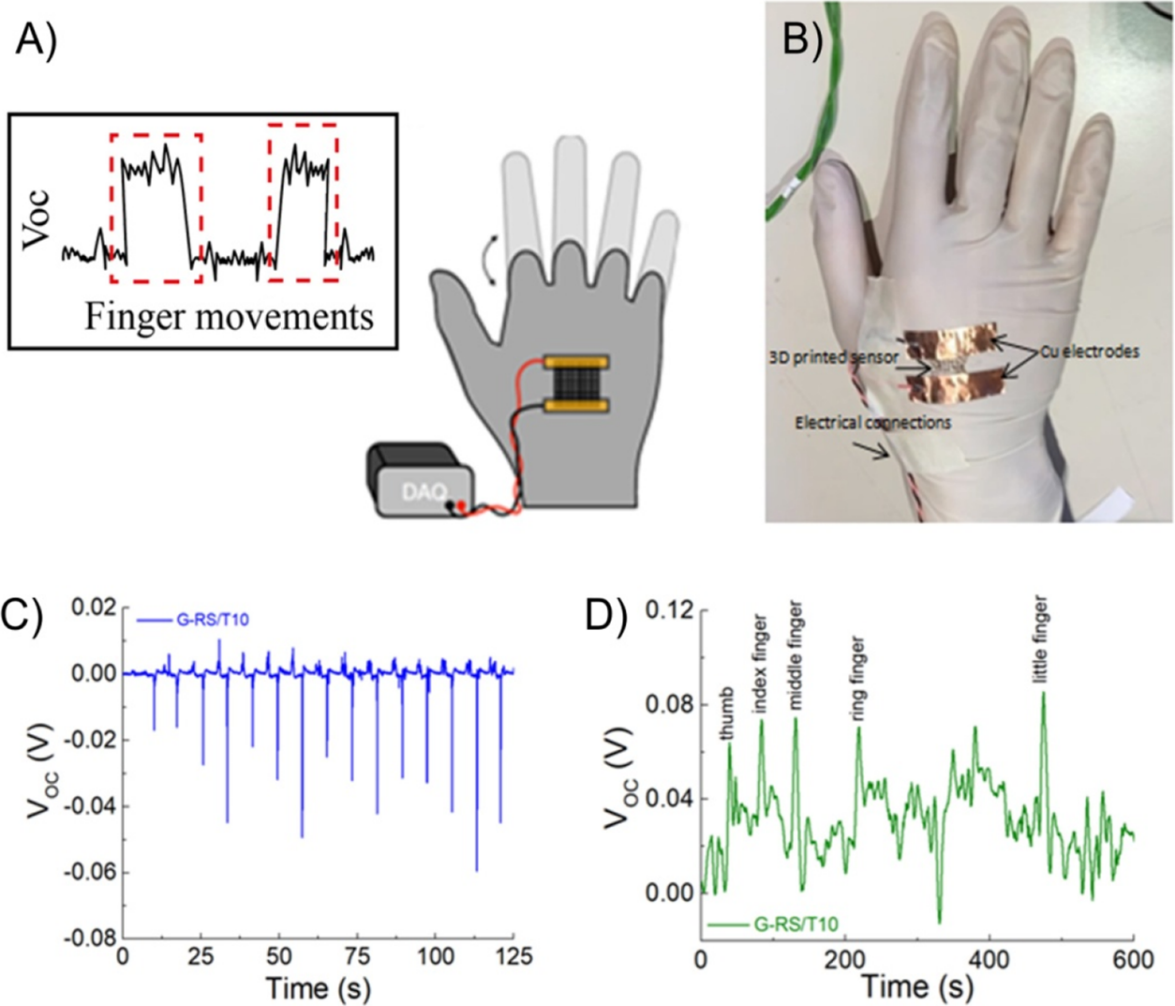
(A) Photographs showing various associated hand gestures and (B) smart glove setup; (C) associated open-circuit voltage response from cyclic strength gesture; and (D) finger movements from the G-RS/T10 printed grid.

Finally, we have exploited the grids as sensors for monitoring complex hand movements, exploring possible applications for sign language translation, and as a rehabilitation tool (Figure 12). The flexibility and conformability of our grids allowed us to fit the anatomy of the hand, while the self-adhesive properties stabilize the sensors into the glove for long-term usages. The smart glove as a prototype device is reported in Figure 12A,B. Also, in this test, the grid was electrically connected to the DMM using cables soldered to Cu adhesive strips, fixating the cables such as to minimize triboelectric or cable-related effects. The glove discerns different signs, including strength and finger movements. In Figure 12C,D, we reported the DC signals of the various hand gestures in different configurations [*i.e.*, strength in Figure 12C (Figure S3) and finger movements in Figure 12D].

## 4 Conclusions

The obtained results span topics in material formulations, processing, and *in vitro* evaluation of self-adhesive bioresorbable materials envisioning applications in wearable electronics and implantable devices. In this paper, we used silk fibroin in FA-soluble plant-derived polyphenols as a biomaterial ink for the preparation of biocompatible 3D printed multilayered holey structures. The self-adhesive nature and the flexibility of these structures maximize their application on soft substrates. Moreover, 3D printing on a water-soluble substrate allowed us to easily and safely transfer the device on the desired substrate without the use of external forces, heat, or tools. The findings include demonstrations of the 3D printed sensors with output open-circuit voltages that demonstrate the coupling of the mechanical deformations and piezoelectric effects as a function of their composition. The proofs of concepts developed in this study are an important starting point for the development of advanced biotic–abiotic interfaces to support diagnosis and treatment in medical applications.

## Supporting Information Available

Current–voltage characteristics of the G-RS sample prepared with different graphene concentrations; mechanical properties of prepared films; open-circuit voltage response from cyclic strength gesture from the RS/T1, G-RS/T1, and RS/T10 printed grids; and FE modeling (PDF)
Bursting experiment (MP4)
Test under cyclic conditions of a grid adhered to an inflated latex balloon (MP4)
