# Supplementary material for: 3D Printing Silk-Based Bioresorbable Piezoelectric Self-Adhesive Holey Structures for In Vivo Monitoring on Soft Tissues
Source: ACS Appl Mater Interfaces. 2022 Apr 19;14(17):19253–64. doi: 10.1021/acsami.2c04078 (PMC9073835; doi:10.1021/acsami.2c04078)
Supplement: Supplementary file 1 — am2c04078_si_001.pdf [file am2c04078_si_001.pdf]

Supporting Information

3D Printing Silk-based Bioresorbable Piezoelectric Self-Adhesive Holey Structures for *In Vivo* Monitoring on Soft Tissues

Irene Chiesa,[a] Carmelo De Maria,*[a] Maria Rachele Ceccarini,[b] Lorenzo Mussolin,[c] Riccardo Coletta,[d,e] Antonino Morabito,[d,f] Rodolfo Tonin[g], Martino Calamai[h,i], Amelia Morrone[f,g], Tommaso Beccari[b] and Luca Valentini*[l,m]

[a] Department of Ingegneria dell'Informazione and Research Center E. Piaggio, University of Pisa, Largo Lucio Lazzarino 1, 56122 Pisa, Italy

[b] Department of Pharmaceutical Sciences, University of Perugia, 06123 Perugia, Italy

[c] Department of Physics and Geology, University of Perugia, 06123 Perugia, Italy

[d] Department of Pediatric Surgery, Meyer Children's Hospital, Viale Pieraccini 24, 50139 Firenze, Italy

[e] School of Health and Society, University of Salford, Salford, United Kingdom

[f] Dipartimento Neuroscienze, Psicologia, Area del Farmaco e della Salute del Bambino NEUROFARBA, Università degli Studi di Firenze, Viale Pieraccini 6, 50121 Firenze, Italy

[g] Molecular and Cell Biology Laboratory, Paediatric Neurology Unit and Laboratories, Neuroscience Department, Meyer Children's Hospital, Firenze, Italy

[h] European Laboratory for non-linear Spectroscopy (LENS), University of Florence, Sesto Fiorentino, Italy.

[i] National Institute of Optics - National Research Council (CNR-INO), Sesto Fiorentino, Italy.

[l] Civil and Environmental Engineering Department, University of Perugia, Strada di Pentima 4, 05100, Terni, Italy

[m] Italian Consortium for Science and Technology of Materials (INSTM), Via Giusti 9, 50121 Firenze, Italy

Corresponding author: luca.valentini@unipg.it

Supporting Information

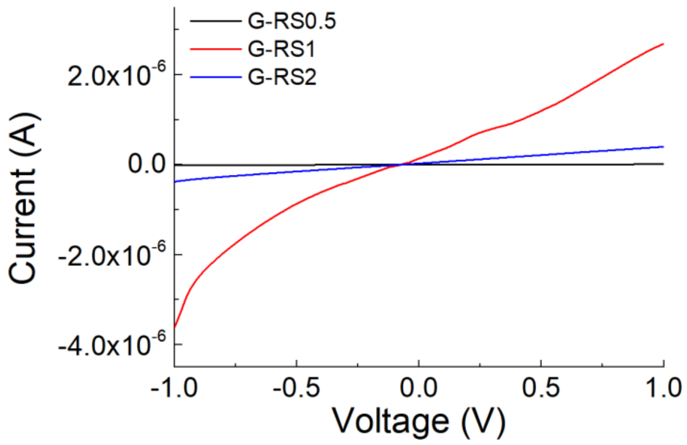

Figure S1. Voltage-current characterization of RS loaded with 0.5 wt% (G-RS0.5), 1 wt% (G-RS1) and 2 wt% (G-RS2), respectively

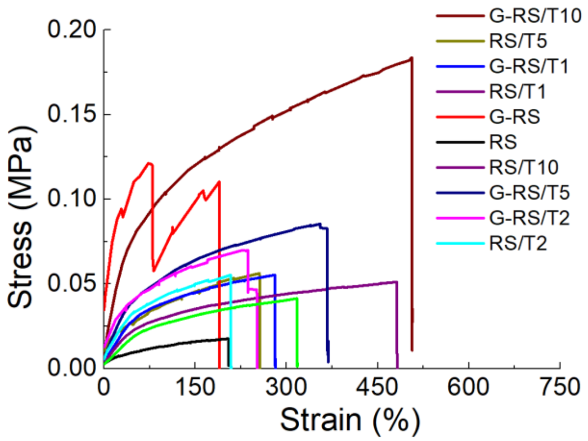

Figure S2. Stress-strain curves of the prepared films.

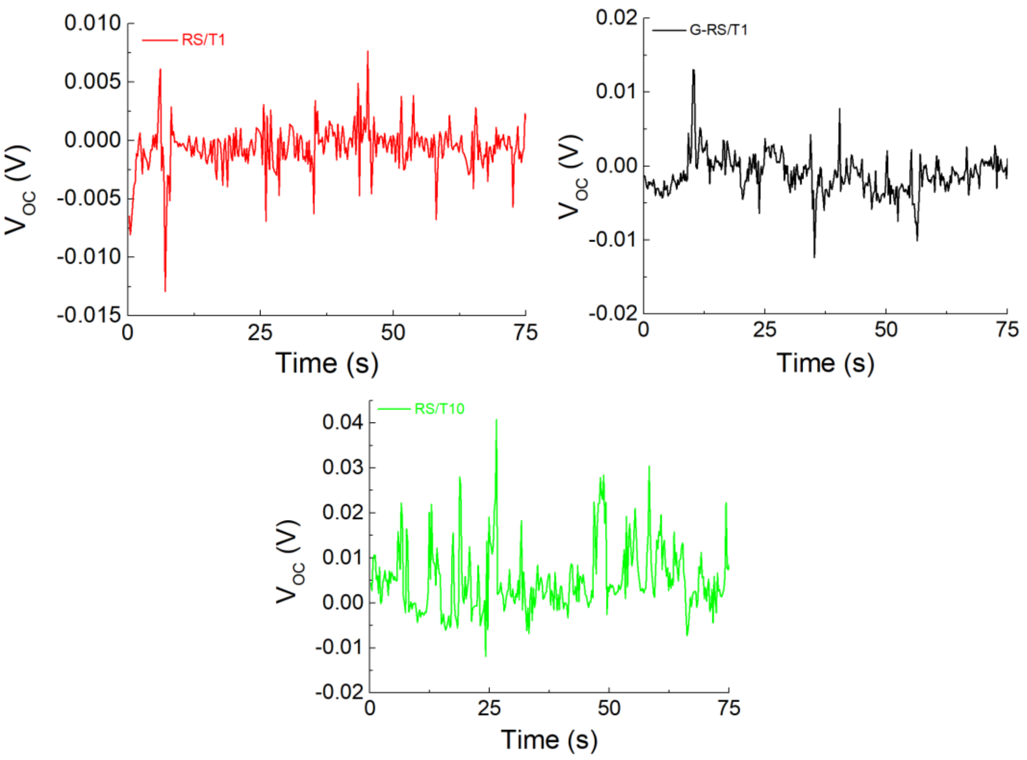

Figure S3. Open circuit voltage response from cyclic strength gesture from the RS/T1, G-RS/T1 and RS/T10 printed grids, respectively.

Supporting Information

Table S1: Mesh statistics of all FE simulations.

	15%	30%	50%	75 %	100%
Maximum element size [mm]	0.0803	0.0803	0.0802	0.0802	0.0803
Minimum element size [mm]	1.61E-4	1.61E-4	1.61E-4	1.61E-4	1.61E-4
Curvature factor	0.2	0.2	0.2	0.2	0.2
Number of elements	12050	17366	21904	27126	24910
Minimum quality	0.6285	0.7075	0.7663	0.709	0.7519
Average quality	0.9508	0.8913	0.968	0.9524	0.9941
